# Brief, Low-impact, High-intensity Osteogenic Loading in Postmenopausal Osteoporosis: A Quasi-experimental Case-series Study

**DOI:** 10.1210/clinem/dgaf077

**Published:** 2025-02-07

**Authors:** Nektaria Papadopoulou–Marketou, Anna Papageorgiou, Nikolaos Marketos, Panagiotis Tsiamyrtzis, Georgios Vavetsis, George P Chrousos

**Affiliations:** Neuroendocrine Tumor Unit, ENETS Centre of Excellence, 1st Department of Propaedeutic and Internal Medicine, Laiko General Hospital, National and Kapodistrian University of Athens, 11527 Athens, Greece; University Research Institute of Maternal and Child Health and Precision Medicine, National and Kapodistrian University of Athens, Athens 11527, Greece; University Research Institute of Maternal and Child Health and Precision Medicine, National and Kapodistrian University of Athens, Athens 11527, Greece; Department of Physiology, Medical School, National and Kapodistrian University of Athens, Athens 11527, Greece; Deptartment of Mechanical Engineering, Politecnico di Milano, Milan 20156, Italy; Department of Statistics, Athens University of Economics and Business, Athens 10434, Greece; University Research Institute of Maternal and Child Health and Precision Medicine, National and Kapodistrian University of Athens, Athens 11527, Greece; University Research Institute of Maternal and Child Health and Precision Medicine, National and Kapodistrian University of Athens, Athens 11527, Greece

**Keywords:** osteostrong, osteoporosis, bone metabolism, exercise in osteoporosis, osteogenic loading, antiosteoporotic treatment

## Abstract

**Background:**

Osteoporosis is characterized by reduced bone mineral density (BMD) and disrupted microarchitecture estimated by trabecular bone score (TBS). Osteostrong^®^ is a bone-strengthening system implementing 4 devices and incorporating brief (10-minute), weekly, low-impact, high-intensity osteogenic loading exercises. The aim of this study was to assess changes in BMD and TBS in the lumbar spine and/or hip over 12 months in peri-/post menopausal women with Osteoporosis following Osteostrong^®^ intervention and to compare these outcomes with women who did not receive such intervention.

**Methods and Subjects:**

A quasi-experimental case-series study in which 147 participants were separated into 2 groups, following informed consent: 75 in group A receiving Osteostrong^®^ (subgroup G1 without and G2 with antiresorptive medication); and 72 in group B without Osteostrong^®^ (subgroup G3 without and G4 with antiresorptive medication). Changes in lumbar spine and hip BMD and/or TBS were assessed at inclusion and 12 months later. Bonferroni-adjusted non-parametric paired tests examined for significant paired mean differences within each subgroup.

**Results:**

After Bonferroni adjustment, significant increases were observed in lumbar spine BMD in G2 (mean paired change: 0.029 g/cm^2^; Bonferroni-adjusted p < 0.001), and G4 (mean paired change: 0.025 g/cm^2^; Bonferroni-adjusted p = 0.05) as well as BMD-total hip left in G2 (mean paired change: 0.028 g/cm^2^; Bonferroni-adjusted p = 0.05). Other within-group changes in femoral neck BMD, total hip BMD, and TBS did not retain significance following Bonferroni correction.

**Conclusion:**

The Osteostrong^®^ intervention showed modest lumbar spine BMD improvements over 12 months; some subgroup effects were significant but not when Bonferroni-adjusted, warranting cautious interpretation and further randomized trials.

At the dawn of the 21st century, health and wellness-directed measures in Western civilization have successfully addressed many problems of everyday life. Nevertheless, an increased fracture risk state, known as osteoporosis, which is characterized by decreased bone mineral density (BMD) and disrupted microarchitecture due to various factors, remains a global health burden. The endpoint of osteoporosis is the low-energy fracture, while pain, immobilization, and disability in general are some of the main reasons for drug development and research on alternative methods to allay or postpone their incidence. Expected fragility fractures per year rise to 4.5 million in the European Union and 1.5 million in the United States, representing, respectively, a $12 to $18 billion expense for the healthcare systems, with a previous report projecting even higher costs in the years to come (1, 2). Almost three-quarters of these fractures affect postmenopausal women. This makes the prevention of osteoporotic fractures a global priority for healthcare systems.

Apart from standard dual-energy x-ray absorptiometry (DXA), which is currently considered the gold standard for evaluating BMD, measurement of the trabecular bone score (TBS) that consists of a grey-level textural measurement typically obtained from conventional lumbar spine DXA-BMD images, serves as a validated index of bone microarchitecture that is correlated with the mechanical properties of the bone (3). TBS has been added to the already established methods of fracture risk assessment for predicting fracture risk (3). According to the World Health Organization (WHO), osteoporosis is defined by a T-score of −2.5 or lower at the femoral neck, based on reference data from white women aged 20-29 years, as established by the NHANES III database (4). In clinical practice, a diagnosis of osteoporosis in postmenopausal women and in men aged 50 years and older may also be made if the T-score at the lumbar spine, total hip, or femoral neck is −2.5 or lower. The consensus 2023 from International Society of Clinical Densitometry (ISCD) recommends that regions of interest for diagnosis of osteoporosis include lumbar spine, total hip and/or femoral neck (with recommendation to use the lowest T-score from the femoral neck or total hip) (5). A recent study underscored the clinical relevance of discordances in bone mineral density (BMD) measurements between the femoral neck and other proximal femur regions, particularly the trochanter and total hip. Although BMD measurements at the trochanter and total hip are routinely obtained alongside femoral neck assessments, their independent predictive value for fracture risk is often underrecognized. The data presented suggest that discordant T-scores-particularly when total hip or trochanteric BMD is lower than femoral neck BMD—are associated with significantly increased fracture risk, independent of FRAX-based probabilities that rely solely on femoral neck BMD (6). The biological explanation of this observation remains unclear but may relate to regional variations in trabecular and cortical bone composition, or differential susceptibility to measurement artifacts, particularly in the femoral neck, which is prone to osteoarthritic changes. Measurement error, inversely proportional to the region's size, may also contribute, with the femoral neck being more susceptible due to its smaller area, therefore the authors highlighted the potential value of incorporating total hip and/or trochanteric BMD into fracture risk assessments (6).

Despite the plethora of drugs used to treat and/or delay osteoporosis, very few nonpharmaceutical methods have been tested and even fewer have succeeded. Until recently, the treatment of osteoporosis in the setting of bone fractures, especially those at the lumbar spine level, consisted of pharmacotherapy, surgery, and the avoidance of everyday exercise, with controversial results. The literature describes nonpharmaceutical methods to reduce fracture risk due to osteoporosis as traditional, complementary, and integrative medicine (7). Patients often use these methods without informing their physicians, making it even more difficult to evaluate the possible protective effects of these practices against osteoporosis (7). Among these proposed methods, supplementation of calcium and vitamin D, along with exercise, remain the most often recommended modality for osteoporosis prevention according to worldwide clinical practice guidelines, although specific parameters of exercise have not been defined (7-9).

There is substantial patient interest in nonpharmacologic approaches to treating or preventing osteoporosis, and Osteostrong^®^ is one such intervention, whose efficiency, however, has not been evaluated in clinical trials yet. Interestingly, suggestions for exercise to strengthen the musculoskeletal system and to increase balance have been provided in recent treatment guidelines for osteoporosis and fracture prevention, without specific instructions for the duration, extension, type of exercise, and other important parameters (10, 11).

Osteogenic loading is based on Wolff's law suggesting that a healthy animal's bone will adjust to the forces applied to it (12). The internal structure of trabeculae experiences adaptive alterations, which are subsequently followed by secondary adjustments to the external cortical part of the bone, processes that could result in the thickening of the bone. Conversely, when the load on a bone is reduced, the bone experiences a decrease in density and strength due to the absence of the necessary stimulus required for continuous remodeling (13-15). Osteostrong^®^, a brief, low-impact, high-intensity osteogenic loading training modality with once-weekly, 10-minute sessions, using patented devices, known as “Spectrum,” is described here. Although several people have used the Osteostrong^®^ intervention with reportedly beneficial effects on bone density, the method has been less studied for its efficacy in improving bone density and quality in subjects with osteopenia or osteoporosis. The aim of this prospective quasi-experimental case-series study was to assess changes in BMD and TBS in the lumbar spine and/or hip over 12 months in peri-/postmenopausal women with osteoporosis following Osteostrong^®^ intervention, and to compare these outcomes with those in women who did not receive the intervention.

## Methods and Study Population

### Osteostrong^®^ Method

Osteostrong**^®^** consists of a series of brief, 10-minute total, weekly exercises of osteogenic loading tailored specifically for every person to ameliorate osteoporosis. This exercise is facilitated using 4 different apparatuses and is characterized by low impact but high intensity, making it more attractive for patients to adhere to and to avoid possible risks of lumbar or other injury, especially in the elderly and individuals with more severe osteoporosis. This equipment applies a certain dosage of force specific to the participants' multiples of bodyweight (MOB), allowing for an increased likelihood of osteogenesis. Spectrum devices emulate these forces in a safe and controlled environment. Designed to enable users to assume optimal leverage positions, each device allows high-force application in static postures, aiming to achieve bone-strengthening impact without actual high-impact activity, taking advantage of the individual's physiological strength limit and preventing injury. The 4 Spectrum devices are based on Growth Trigger (GT), indicating each device''s objective to facilitate bone adaptation (Upper GT, Lower GT, Postural GT, and Core GT), each focusing on distinct regions of the musculoskeletal system. The Upper GT targets the kinetic chain from hands to clavicle; the Lower GT from feet to hip; the Postural GT from feet to neck; finally, the Core GT focuses on the rib cage. Moreover, the Osteostrong^®^ intervention program includes falls prevention and balance training exercises monitored by physiotherapists, with a focus on posture, stability, mobility, and coordination.

### Study Population

A total of 147 peri-/postmenopausal women with osteoporosis of the lumbar spine and/or femoral neck or total hip, who were followed at and recruited from the Unit on Clinical and Translational Research in Endocrinology, National and Kapodistrian University of Athens, Greece, were enrolled in this quasi-experimental case-series prospective study. Recruitment began concurrently with enrollment on April 1, 2022. All participants were Caucasian adult women of Greek origin.; as of 1st April 2022, they were introduced into the study protocol and monitored closely by endocrinologists till the end of their study, 12 months after the onset. The study was approved by the Ethical Committee of the University Research Institute (URI) of Maternal and Child Health and Precision Medicine (former title: URI for the Study and Treatment of Genetic and Malignant Disorders of Childhood), Athens, Greece. A consent form was obtained from all the participants. Inclusion criteria were female gender, peri-/menopause that presented either early or at the expected age, and osteoporosis measured by DXA in either the lumbar spine and/or, hip. and femoral neck. The three sites of measurements were decided, considering the clinical relevance of discordances in BMD measurements between the femoral neck and other proximal femur regions, particularly the total hip (5,6).

Exclusion criteria were secondary osteoporosis and recent bone fracture. Patients were assigned to the various treatment subgroups according to their status and preference, after they had been informed about the Osteostrong^®^ intervention. They were first divided into 2 groups comprising two subgroups each. Participants who accepted to receive the Osteostrong^®^ intervention represented the experimental Group A, which included 75 women treated with Osteostrong^®^ (mean age: 58.7y, 95% confidence interval 56.5-60.9); this group was subdivided into subgroup G1 of 53 women, who had no parallel antiresorptive treatment, and G2 of 22 women, who were treated in parallel with either oral bisphosphonates or denosumab. Those who for various reasons (mostly time constraints and convenience) were not interested in receiving or unable to participate in the Osteostrong^®^ intervention served as a parallel control Group B, which included 72 women (mean age 61.8 years, 95% confidence interval 59.4–64.2). Group B was subdivided into subgroup G3 of 35 women who did not receive antiresorptive treatment, and G4 of 37 women who were treated with such medication. Data regarding body mass index and age are shown in Table 1. None of the patients from either subgroups G1 or G3 had previous antiresorptive treatment. In subgroups G2 and G4, denosumab, alendronate, and/or risendronate were the antiresorptive drugs used. For detailed information on subgroups G2 and G4, see Supplementary material (16). None of the participants were on hormone replacement therapy, even though this was not excluded as an option criterion, particularly in those with early menopause. Only one patient from subgroup G1 was diagnosed with early menopause. All patients received calcium and vitamin D supplementation, as indicated. In terms of comorbidities, several participants had medications for conditions such as hypothyroidism, arterial hypertension and/or dyslipidemia.

**Table 1.** Body mass index (BMI) and age in the different subgroups (Subgroups, G1: Osteostrong, without pharmacologic treatment, G2: Osteostrong, with pharmacologic treatment, Group G3: No Osteostrong, nor pharmacologic treatment, G4: No Osteostrong, with pharmacologic treatment).

	G1		G2		G3		G4	
	Mean	SD	Mean	SD	Mean	SD	Mean	SD
Age	57.706	7.862	61.045	12.065	58.781	9.827	64.378	9.364
BMI	24.080	4.714	22.836	2.220	25.418	4.279	25.068	4.020

All participants underwent a complete physical examination and an assessment for exclusion of secondary osteoporosis. Details from medical history, such as uptake of glucocorticoids or previous fragility bone fracture, were retrieved, and laboratory tests to exclude primary hyperparathyroidism, connective tissue disorders, hypercortisolism, vitamin D insufficiency, or other conditions, such as sarcoidosis or blood malignancy, were conducted. DXA examination [Horizon W(S/N 300472M) twice, at the time of inclusion in the trial and 12 months later, was performed. Bone markers [C-terminal telopeptide of type I collagen (CTX-I) and N-terminal propeptide of type I procollagen (P1NP)] were examined after the intervention (the laboratory tests were conducted early in the morning after an overnight fast of 8 hours) in groups G1 and G2.

### Statistical Methods

The power of the study (significance, alpha = 0.01, beta = 0.01, mean difference = 0.03 and SD = 0.02) initially estimated a sample size of 14 for each group; Statistical analysis for each of the recorded response variables involved non-parametric (paired Wilcoxon tests), along with regression analyses. The paired tests examined significant mean differences within each group during the study. The regression analyses assessed whether we had significant differences of each subgroup (G1, G2 and G4) from the baseline subgroup G3 (no antiresorptive therapy and no use of Osteostrong), taking into account the effects of age, BMI and serum vitamin D3 levels. Pairwise comparisons between groups have also been performed (16). When for a response variable either the Start or the End measurement was missing, this subject was not included in the specific test. The exact number of missing values in each subgroup and each test for each of the recorded responses, along with the descriptive statistics of the paired differences End-Start are shown in (16). All analyses were performed using the freeware R (version 4.2.2). The primary endpoints of the study were changes in BMD and/or TBS following the intervention, with favorable outcomes defined as at least stability or improvement in BMD or TBS values.

The statistical analyses addressed the following research questions (RQ):

RQ1: For each of the 12 responses separately, we were interested in testing the following hypotheses:
  H0: mean paired difference (End-Start) = 0
  H1: mean paired difference (End-Start) > 0

We performed this hypothesis test in each of the four groups separately (4 groups x 12 responses = 48 hypothesis tests).

RQ1: Hypothesis Testing in Each Group and Each Response.

**Figure 1.**
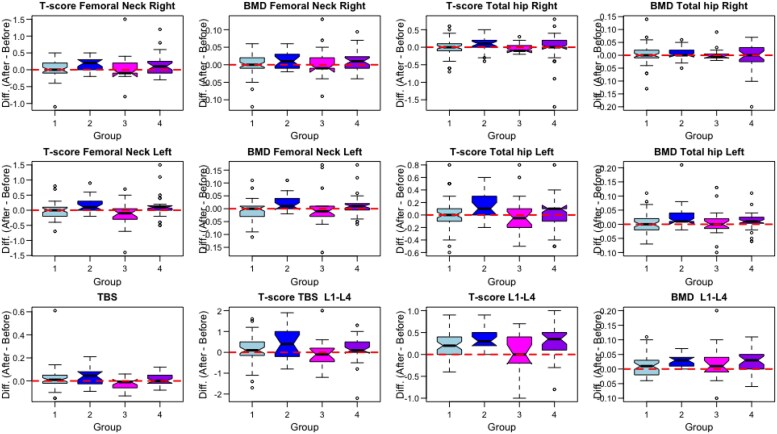
The boxplots display the paired differences (End-Start) between each of the four subgroups and for each of the 12 recorded responses. In each case, a horizontal line at zero is included to denote no-change. (Subgroups, G1: Osteostrong without pharmacologic treatment, G2: Osteostrong with pharmacologic treatment, G3: No Osteostrong, nor pharmacologic treatment, G4: No Osteostrong, with pharmacologic treatment)

We began by providing in Figure 1, the boxplots of the differences (End-Start) for each of the four groups (G1-G4) and for each of the 12 recorded responses, which were used in the non-parametric paired tests (individual value plots of the Start and End points can be found in 16). In each case of Figure 1, a horizontal line at zero denotes the no-change case. In this research question, our goal was to test whether the mean response was the same at the start and end (H0) vs. whether the mean response at the end of the study was higher (H1) within each subject. We performed 48 paired t-tests. In Table 2, we provide test statistics and p-values (unadjusted and Bonferroni adjusted) for the non-parametric (paired Wilcoxon tests). Specifically, in Table 2, the paired t-test p-values, we observed that there were 19 cases where we had significant differences (p-value < 0.05) at End vs. Start. However, when adjusting for multiple testing using Bonferroni's correction, which is known to be extremely conservative, only 6 cases remained significant. The paired T-test parametric approach was also performed and provided very similar results to the non-parametric analysis. The parametric based results along with the respective assumption checking can be found in the online supplementary material (16).

**Table 2.** Non-parametric (paired Wilcoxon test) to examine significant differences between End-Start per subgroup and response. Test statistics along with the unadjusted and Bonferroni adjusted p-values of the non-parametric paired-Wilcoxon tests (p-values below 0.05 are indicated in boldface). Subgroups, G1: Osteostrong without pharmacologic treatment, G2: Osteostrong with pharmacologic treatment, G3: No Osteostrong, nor pharmacologic treatment, G4: No Osteostrong, on pharmacologic treatment. BMD: bone mineral density, TBS: trabecular bone score.

Response	Group	# of complete cases	Non-parametric Paired Wilcoxon test statistic for one sided alternative (End>Start)	Non-parametric Paired Wilcoxon test p-value for one sided alternative (End>Start)	Bonferroni adjusted Non-parametric Paired Wilcoxon p-values = min{1, p-value*48}
**T-score Neck Right**	**1**	45	406	0.127	1
	**2**	21	160.5	**0.004**	0.19
	**3**	18	67.5	0.521	1
	**4**	28	248.5	**0.032**	1
**BMD Neck Right**	**1**	45	461.5	**0.049**	1
	**2**	21	141	**0.008**	0.38
	**3**	17	71.5	0.438	1
	**4**	29	238.5	0.055	1
**T-score Total Right**	**1**	45	307	0.556	1
	**2**	21	97	0.070	1
	**3**	16	44.5	0.704	1
	**4**	29	128	0.338	1
**BMD Total Right**	**1**	44	317.5	0.256	1
	**2**	21	69	0.053	1
	**3**	16	35	0.640	1
	**4**	28	95	0.653	1
**T-score Neck Left**	**1**	50	339	0.832	1
	**2**	22	159	**0.005**	0.24
	**3**	20	50	0.900	1
	**4**	31	185.5	0.076	1
**BMD Neck Left**	**1**	50	407	0.897	1
	**2**	22	142	**0.007**	0.34
	**3**	20	55.5	0.847	1
	**4**	31	233.5	0.072	1
**T-score Total Left**	**1**	50	293	0.738	1
	**2**	22	135.5	**0.003**	0.14
	**3**	20	68	0.665	1
	**4**	31	221.5	0.221	1
**BMD Total Left**	**1**	50	383.5	0.805	1
	**2**	21	157	**0.001**	**0.05**
	**3**	20	78	0.481	1
	**4**	31	332	**0.020**	0.96
**TBS**	**1**	45	555	0.099	1
	**2**	20	134.5	**0.017**	0.82
	**3**	16	22	0.974	1
	**4**	29	173	0.147	1
**T-score TBS L1 L4**	**1**	47	639	**0.047**	1
	**2**	18	126	**0.01**	0.48
	**3**	16	35	0.779	1
	**4**	29	181	**0.040**	1
**T-score L1 L4 Total**	**1**	50	904.5	**<0.001**	**0**
	**2**	21	190	**<0.001**	**0**
	**3**	18	57.5	0.21	1
	**4**	30	313	**<0.001**	**0**
**BMD Total L1 L4**	**1**	48	701.5	**0.019**	0.91
	**2**	21	171	**<0.001**	**0**
	**3**	17	83.5	0.095	1
	**4**	31	346.5	**0.001**	**0.05**

RQ2: 12 Regression Models (One for Each Response) vs. Group, Age, BMI and Vitamin D3.

For both response variables, we calculated the difference between the two time point measurements: End-Start, indicating the changes in the recorded response variable over 12 months. Using a regression model with the difference of each response as the dependent variable and predictors including Group, Age, BMI and serum Vitamin D3, we examined whether there were significant differences in groups G1, G2, and G4 compared to the baseline G3 (i.e., whether the mean difference in any of these groups was significantly different from the mean differences in the baseline group) and whether Age, BMI and Vitamin D played any significant role. We ran the regression model using the End-Start difference measurements for each response (i.e., using a single number for each subject), testing for significance between groups G1, G2 and G4 vs. the G3, which acted as the baseline, indicating no use of Osteostrong and no medication, along with Age, BMI and Vitamin D3. In Table 3, we provided the coefficients and p-values of the predictors in each of the 12 models (one for each response). There were seven cases where we found statistical significance (i.e., p value < 0.05), but only three remained significant after Bonferroni correction (i.e. p-value<0.0083). The Bonferroni adjusted significant factors refer to (G2 vs. G3) and (G4 vs. G3) in T-score Femoral Neck Left and (G2 vs. G3) in T-score L1-L4. All the statistical analyses can be retrieved in the supplementary material (16).

**Table 3.** The coefficients and p-values of the regression model applied for each response separately (p-values below 0.05 are indicated in boldface). Subgroups, G1: Osteostrong without pharmacologic treatment, G2: Osteostrong with treatment, Group G3: No Osteostrong, nor pharmacologic treatment, G4: No Osteostrong, pharmacologic treatment. BMD: bone mineral density, TBS: trabecular bone score).

	G1 vs G3		G2 vs G3		G4 vs G3		BMI		Age		25 OH Vitamin D3(end)	
Response	Coeff.	p-value	Coeff.	p-value	Coeff.	p-value	Coeff.	p-value	Coeff.	p-value	Coeff.	p-value
**T-score Femoral Neck Right**	-0.071	0.566	0.042	0.756	0.045	0.742	0.001	0.899	0.005	0.24	-0.004	0.536
**BMD Femoral Neck Right**	-0.007	0.619	0.003	0.829	0.005	0.726	-6E-04	0.588	2E-04	0.635	-6E-04	0.362
**T-score Total hip Right**	0.057	0.651	0.073	0.59	0.055	0.691	-0.015	0.141	-0.003	0.468	-0.009	0.143
**BMD Total hip Right**	0.003	0.86	0.003	0.839	-0.013	0.444	-0.002	0.22	-6E-05	0.923	-0.002	**0.047**
**T-score Femoral Neck Left**	0.201	0.101	0.38	**0.005**	0.407	**0.003**	-3E-04	0.98	-0.008	0.088	-0.013	**0.039**
**BMD Femoral Neck Left**	-0.004	0.799	0.024	0.215	0.026	0.177	6E-04	0.673	-1E-03	0.165	-6E-04	0.469
**T-score Total hip Left**	-0.016	0.85	0.063	0.503	0.016	0.868	-0.006	0.46	0.002	0.615	-0.008	0.077
**BMD Total hip Left**	-0.002	0.852	0.013	0.36	0.017	0.244	-1E-04	0.909	-5E-04	0.319	-0.001	0.114
**TBS**	0.039	0.241	0.053	0.134	0.015	0.684	5E-05	0.986	0.003	**0.014**	0.001	0.506
**T-score TBS L1 -L4**	-0.101	0.661	0.245	0.338	0.116	0.655	0.015	0.408	0.013	0.145	-0.003	0.751
**T-score L1-L4**	0.168	0.115	0.333	**0.005**	0.254	**0.034**	0.003	0.718	0.002	0.606	-5E-04	0.916
**BMD L1- L4**	-0.006	0.7	0.006	0.718	0.005	0.755	-0.001	0.3	-2E-05	0.964	-0.001	0.117

## Results

The main adverse event that was feared by the participants and closely monitored by the Osteostrong® facility personnel was a new bone fracture at any site and/or a muscle or tendon trauma during the procedure. No such events occurred in any participant from G1 and G2. In total, 13 patients experienced joint pain, and 3 patients (two from G1 and from G2) dropped out of the study because of it. One patient from G3 and 1 from G4 experienced a lumbar spine fracture. BMD measurements at baseline and 12 months later are shown in Tables 2-4. Statistical analyses utilizing paired Wilcoxon tests (both unadjusted and Bonferroni adjusted) were conducted on all parameters measured before and after the Osteostrong^®^ intervention. The results indicate the following findings:

***Lumbar spine*** (L1-L4): After 12 months, significant increases in lumbar spine BMD were observed only in subgroups G2 (n=21) and G4 (n=31) following Bonferroni correction. In G2 (Osteostrong^®^ + antiresorptive), the mean lumbar spine BMD increase was 0.029 g/cm^2^ (Bonferroni-adjusted p < 0.001), while in G4 (antiresorptive only), the increase was 0.025 g/cm^2^ (Bonferroni-adjusted p = 0.05). G1 showed a modest increase (non-parametric p:0.019, mean change: 0.011 g/cm^2^; Bonferroni-adjusted p = 0.91).
***TBS***: An increase in TBS was observed in subgroup G2 (n = 20), with values of non-parametric p-value: p = 0.017 (Bonferroni adjusted p = 0.828); mean difference for End-Start was 0.039.
***Right femoral neck***: In subgroup G2 (n=21), BMD was increased (non-parametric p=0.008, Bonferroni adjusted p = 0.389); respective mean End-Start difference was 0.013 gram/cm^2^.
***Left femoral neck***: In subgroup G2 (n = 22), an increase in BMD was seen (non-parametric p=0.007; Bonferroni adjusted p = 0.34), with a mean difference for End-Start of 0.02 gram/cm^2^.
***Left total hip:*** In subgroup G2 (n = 21), an increase in BMD was seen (non-parametric p = 0.001; Bonferroni adjusted p = 0.05), with mean difference for End-Start recorded as 0.028 gram/cm^2^. In G4 (n = 31), the BMD was increased (non-parametric p = 0.02; Bonferroni adjusted p = 0.96), with a mean End-Start difference of 0.012 gram/cm^2^.
***Right total hip:*** No statistically significant changes were found.

T-scores changes for all the measurements are shown in Table 2 (16).

All data are illustrated in Figure 1 and Tables 2 through 4 and in the online supplementary material (16). Descriptive statistics of the paired End-Start differences per subgroup, including percentages of positive, negative, and zero changes, are provided in supplementary material (16). Regression analyses assessing the influence of age and body mass index were performed; however, no statistically significant relationships were observed (see Table 3). For patients in G2 and G4 receiving Denosumab, a regression analysis concerning changes in lumbar spine TBS was conducted. Although a trend towards increased TBS was noted, statistical significance was not achieved. Among patients in G3 who received neither the Osteostrong^®^ intervention nor antiresorptive treatment, no statistically significant change was found. Lastly, no statistically significant correlations were detected between P1NP or CTX-I levels and changes in BMD across any of the groups.

## Discussion

This study is the first to investigate the potential effects of the Osteostrong^®^ intervention on BMD and TBS in peri-/postmenopausal women with osteoporosis. Statistically significant increases in lumbar spine BMD were observed in participants receiving the intervention, particularly in those who were also receiving antiresorptive therapy. However, after Bonferroni correction for multiple comparisons, only a subset of these findings remained statistically significant. Improvements in TBS were observed in the subgroup receiving both Osteostrong^®^ and pharmacologic treatment, though these did not retain significance after Bonferroni correction either. Small increases in BMD and T-score at the femoral neck and hip were also noted in intervention groups, but again, few reached significance when adjusting for multiple comparisons. In contrast, participants who did not receive any intervention tended to show minor, nonsignificant changes in the evaluated bone parameters.

Osteoporosis reflects the risk of bone fracture, often resulting in pain, surgery, and complications owing to prolonged hospitalization and workforce loss. Osteoporosis is a global socioeconomic burden that affects millions of people and is associated with a low quality of life. Despite the administration of numerous treatment modalities over the years, no nonpharmacological approach has demonstrated satisfactory results.

Literature has extensively discussed various exercise modalities and their effects on bone mass and, eventually, osteoporosis. The notion of a “mechanostat” on bone was introduced by Wolff and then described in detail by Frost (12-15). The idea of a bone sensor that responds positively to external forces through muscle training, resulting in improved thickness and microarchitecture, has been compelling but never shown in a clinical trial. This is the first time that the administration of training in a calculated and individually tailored manner has been studied for its effect on the BMD. The lack of contraindications to this treatment modality, as it does not interfere with any of the medications employed, makes it more attractive irrespectively of age, socioeconomic status, and comorbidities, resulting in strong adherence to an exercise plan.

Earlier studies have shown a positive effect of low-load, high-repetition resistance exercise on the lumbar spine BMD of otherwise healthy postmenopausal women compared to controls who did not exercise (17). Although the beneficial effect of progressive resistance training on the lumbar spine was not indicated in any of the literature reviews (18), perhaps because of the large variation in the studies included, the approach of low-intensity, high-repetitive exercise tended to be abandoned as more recent evidence emerges. Alternative methods of training, such as Tai Chi Chuan, have also shown beneficial effects on lumbar spine BMD (19), but availability is certainly an issue. In men with osteopenia, high-intensity resistance and impact training (HiRIT) has shown a beneficial effect on bone geometry and strength in the femoral neck compared to isometric training based on machines (20). Similarly, stable values for lumbar spine BMD after 12 months of high-intensity exercise were shown in men with osteosarcopenia according to the Franconian Osteopenia and Sarcopenia Trial (21).

Previous studies have shown that osteogenic loading positively affects spinal osteoporosis. HiRIT has been assessed previously in postmenopausal women with encouraging results (19-26). Additionally, the high-intensity exercise implemented not only did not cause extra-vertebral fractures but also improved thoracic kyphosis in this group of patients (19, 23, 24). Moreover, HiRIT was associated with greater improvement in BMD of the lumbar spine of osteoporotic postmenopausal women than with medium-intensity resistance and impact training, thus diminishing the fear of new fractures in this vulnerable population, despite the small number of clinical trials evaluating HiRIT (26-29).

According to the Preferred Reporting Items for Systematic Reviews and Meta-Analyses statement (30), literature reviews based on (i) controlled trials, (ii) isolated Dynamic Resistance exercise (DRT) with at least 1 exercise and 1 control group, (iii) intervention durations ≥6 months, (iv) BMD assessments at the lumbar spine or proximal hip, and (v) cohorts of postmenopausal women, revealed that once-weekly exercise benefitted postmenopausal women with osteoporosis in comparison to more frequent exercise concerning lumbar spine BMD and was the sole parameter that could be suggested regarding the modus of exercise (28, 29, 31-33). Similar positive results for lumbar spine BMD after high-intensity exercise were reported in another review further supporting this argument (34). Moreover, positive effects of high-intensity exercise on lumbar spine BMD in postmenopausal women with osteopenia or osteoporosis were also shown in the ACTLIFE-RCT trial (35). Nevertheless, it is difficult to address all parameters needed for optimal outcomes through exercise. Even when classified according to the type of exercise (weight-bearing, dynamic resistance, and mixed interventions), no clear differences were observed, as all were beneficial for the lumbar spine BMD of postmenopausal women (32). A recent review and meta-analysis isolated 14 of 780 studies concerning the mode (resistance only vs. combined resistance and weight-bearing exercises), frequency, volume, load, and program length (36). It was shown that increases in BMD were favored by combined resistance and weight-bearing exercises, lower volumes, and higher loads, although lumbar spine BMD did not benefit from current resistance exercise programs, leaving room for improvement in this domain (36). To address the difficult aspects of the optimal resistance-training modality, a new trial is currently underway (37).

The beneficial effect of progressive resistance training with specifically tailored, multicomponent exercise programs, along with other health adjustments for balance and nutrition, have been recommended for the management of patients with osteoporosis. Implementation of these encouraging results fueled the recent UK guidelines. Resistance and impact training is recommended for patients with osteoporosis; however, it is clearly stated that the benefit of physical activity often outweighs the risk of harm (31). Nevertheless, a more careful approach regarding the intensity of exercise was adopted because of the lack of evidence of the beneficial effect of high- vs moderate-intensity training.

Exercise recommendation for osteoporosis is often a sensitive matter, especially in the lumbar spine, as the incorrect application of force by the individual may result in new fractures, causing more pain and disability. Osteostrong^®^, which consists of a series of brief, 10-minute, weekly exercises of osteogenic loading tailored specifically for every person to ameliorate osteoporosis, showed opposite results. It was well tolerated by patients of different ages and socioeconomic status who followed this program for an entire year. Additionally, despite the intensity of the exercise, no adverse events, such as new fractures, muscle trauma, or other injuries were reported, thus enhancing patient commitment to the exercise program. This exercise is facilitated using 4 different apparatuses and is characterized by low impact but high intensity, making it more attractive for patients to adhere to and avoid possible risks of lumbar injury, especially for the elderly and individuals with more severe osteoporosis. A previous pilot study on just 1 subject, who was an astronaut, of weekly 15-minute exercise for 6 months showed an increase in body strength but no other osteoporosis parameters, perhaps due to the short period of assessment (38, 39). Similar results were reported from another group, as HiRIT showed a beneficial effect in total hip, femoral neck volumetric, and geometric section modules in comparison to a low-intensity Pilates-based exercise. This effect was further augmented in patients who received antiresorptive treatment in addition to HiRIT (27).

The beneficial results of exercise in diminishing fracture risk, the main endpoint of all antiosteoporotic interventions, was observed in a retrospective, observational study of men (40). This study demonstrated that vigorous but not moderate exercise resulted in a decreased hazard ratio for fracture risk in lifelong athletes, and this effect was progressively more evident with passing years. These results encourage the future application of Osteostrong^®^ principles in other groups of individuals vulnerable to osteopenia or osteoporosis.

Our study had several limitations, including its lack of randomization and quasi-experimental case-series nature, which expose the findings to potential confounding due to group allocation, as differences in baseline characteristics could affect the results. Not all patients who were asked to participate in the study agreed to do so, citing various reasons, such as difficulty adhering to weekly appointments due to distance, work schedules, lack of time, other responsibilities, and fear of trauma. Because the Osteostrong^®^ facilities was only available at a single center in Greece, participation was inherently self-selected. This self-selection bias is exacerbated by the fact that access to the center depended on participants' ability to travel across Athens, potentially skewing the sample toward those with greater mobility and resources. Those who were more motivated or able to commit to the intervention might have had higher baseline physical activity levels, thus they may not represent the broader population of peri-/postmenopausal women with osteoporosis. Furthermore, three patients failed to complete the study for the entire year of treatment, even though no skeletal trauma was observed in any of them. Additionally, some serum parameters, such as CTX-I and P1NP concentrations, were not obtained from all participants and not at the time points of the intervention. The reasons for these missing values varied and included participant withdrawal due to unforeseen circumstances, non-compliance, and potential logistical issues regarding follow-up appointments.

To ensure that the analysis remained robust, subjects with missing data were excluded from specific non-parametric analyses, as indicated in Table 2 and in the Supplementary material (16). Consequently, only complete cases were included, which could impact the interpretation of results and generalizability of findings within this group. Furthermore, we acknowledge that the high percentage of missing data in Group G3, which primarily stems from participants undergoing their second DXA scan on a different machine where TBS was not assessed, than their initial scan and therefore the results were not entirely comparable. In certain cases, the results were difficult to explain. For example, in some individuals, regardless of group assignment, BMD gains were observed in one hip while no change or even loss was seen in the other. While measurements were taken using the same DXA machine to reduce variability, inherent measurement errors still exist in DXA assessments. Factors such as patient positioning and machine calibration may introduce variability in the BMD and TBS results. Although monitoring by endocrinologists was sustained throughout the study, we cannot ensure complete compliance regarding medication intake. Despite efforts to control for confounding variables, such as age, BMI, and pre-existing medical conditions, unaccounted confounders could still influence the outcomes. Additionally, variations in physical activity outside of the intervention or differences in overall health status may have affected BMD and TBS measurements. Another limitation of the study was the variability in baseline diagnostic sites for osteoporosis, as participants had T-score values consistent with osteoporosis at the lumbar spine, femoral neck, or total hip. This heterogeneity contributed to discrepancies in BMD measurements and group-level mean differences at baseline and follow-up, as seen in Table 4. A higher number of participants were initially recruited into G3; however, due to missing data-primarily resulting from follow-up DXA scans being conducted on different machines that did not support TBS assessment-several measurements were excluded from the statistical analysis. In addition, factors such as age, higher BMI or coexisting conditions like osteoarthritis may have contributed to higher BMD values observed in this group.

**Table 4.** Statistics in parameters measured at baseline and 12 months later. Abbreviations: Mean=mean values, SD=standard deviation, Paired Diffs= subject based paired differences within each group, with G1: Osteostrong without pharmacologic treatment, G2: Osteostrong with pharmacologic treatment, G3: No Osteostrong, nor pharmacologic treatment, G4: No Osteostrong, on pharmacologic treatment, BMD: bone mineral density, BMD L1-L4: BMD of the lumbar spine 1 to 4, BMD neck=BMD of the femoral neck, BMD total= BMD of the total femur, TBS=Trabecular bone score, BMD= bone mass index. Boldface entries indicate Bonferroni adjusted significant paired mean differences when the non-parametric paired-Wilcoxon test was applied (see Table 2).

	Mean G1	SD G1	Mean G2	SD G2	Mean G3	SD G3	Mean G4	SD G4
**BMD L1-L4 (Start)**	0.815	0.085	0.768	0.103	0.87	0.107	0.817	0.123
**BMD L1-L4 (End)**	0.821	0.088	0.800	0.104	0.856	0.114	0.844	0.121
**BMD L1-L4 (Paired Diffs)**	0.011	0.033	**0.029**	0.02	0.022	0.065	**0.025**	0.036
**BMD Femoral Neck Left (Start)**	0.642	0.074	0.600	0.063	0.655	0.082	0.608	0.082
**BMD Femoral Neck Left (End)**	0.636	0.070	0.621	0.068	0.682	0.102	0.613	0.087
**BMD Femoral Neck Left (Paired Diffs)**	-0.006	0.036	0.020	0.033	-0.001	0.073	0.013	0.046
**BMD Femoral Neck Right (Start)**	0.642	0.071	0.600	0.065	0.664	0.084	0.625	0.081
**BMD Femoral Neck Right (End)**	0.645	0.069	0.611	0.065	0.683	0.100	0.634	0.086
**BMD Femoral Neck Right (Paired Diffs)**	0.005	0.034	0.013	0.023	0.005	0.048	0.011	0.03
**BMD Total hip Left (Start)**	0.752	0.085	0.708	0.073	0.771	0.086	0.744	0.09
**BMD Total hip Left (End)**	0.752	0.077	0.739	0.059	0.790	0.110	0.744	0.104
**BMD Total hip Left (Paired Diffs)**	-0.001	0.035	**0.028**	0.047	0.003	0.051	0.012	0.034
**BMD Total hip Right (Start)**	0.758	0.077	0.724	0.063	0.77	0.099	0.747	0.08
**BMD Total hip Right (End)**	0.757	0.078	0.736	0.071	0.777	0.094	0.739	0.09
**BMD Total hip Right (Paired Diffs)**	0.002	0.04	0.009	0.025	0.002	0.027	-0.008	0.054
**TBS (Start)**	1.236	0.096	1.18	0.123	1.277	0.085	1.215	0.087
**TBS (End)**	1.254	0.13	1.218	0.099	1.242	0.125	1.215	0.073
**TBS (Paired Diffs)**	0.021	0.108	0.039	0.076	-0.027	0.053	0.013	0.052

The study focused on a specific demographic (Caucasian peri-/postmenopausal women from a particular geographic region), which may limit the generalizability of the findings to more diverse populations of women or men. This demographic specificity may affect the applicability of the results to broader populations. Additionally, the 12-month follow-up period may not have been sufficient to capture long-term effects of the Osteostrong^®^ intervention. Bone remodeling is a dynamic process, and longer durations may be necessary to observe sustained changes in bone density and quality. While the focus on BMD and TBS is crucial, the study could benefit from a broader evaluation of the intervention's impacts on related outcomes, such as quality of life, functional mobility, and incidence of falls, which can influence fracture risk.

On the other hand, the study had certain advantages. The patients were recruited from both the public and private medical sectors, reflecting a socioeconomically balanced group. BMD and TBS measurements were performed on the same DXA machine in all patients whose parameters were included in the statistical analyses, decreasing variability and enhancing the validity of the results. Moreover, patients were referred from the entire Athens metropolitan area to the same principal investigators, who screened, evaluated and monitored all participants using a consistent approach. Additionally, as a quasi-experimental case-series and prospective study, we continue to monitor participants and collect data yearly to assess whether the changes in BMD and/or TBS are temporary or more permanent in individuals who continue or discontinue these exercises.

In conclusion, this prospective case-series study suggests that the Osteostrong^®^ intervention may be associated with modest improvements in lumbar spine BMD, in women with postmenopausal osteoporosis. While unadjusted analyses indicated several statistically significant changes in BMD and TBS across subgroups, only a limited number of these findings remained significant after Bonferroni correction, which was applied to control for multiple hypothesis testing. Notably, the combination of Osteostrong^®^ and antiresorptive medication was associated with greater numerical improvements, particularly in TBS and hip BMD, although these differences did not consistently reach statistical significance after adjusting for the Age, BMI and Vitamin D level covariates. These results highlight the need for cautious interpretation and support the rationale for future randomized, controlled studies in more diverse populations to assess the reproducibility, clinical significance, and potential role of osteogenic loading interventions in osteoporosis management.

## Data Availability

The data supporting the findings of this study are available within the article and online supplementary material (16).
