# Correction to: “Effective Brief, Low-impact, High-intensity Osteogenic Loading in Postmenopausal Osteoporosis”

**DOI:** 10.1210/clinem/dgaf354

**Published:** 2025-06-24

This is a correction to Papadopoulou–Marketou N, Papageorgiou A, Marketos N, Tsiamyrtzis P, Vavetsis G, Chrousos GP, Effective Brief, Low-impact, High-intensity Osteogenic Loading in Postmenopausal Osteoporosis, *The Journal of Clinical Endocrinology & Metabolism*, 2025; dgaf077, https://doi.org/10.1210/clinem/dgaf077.

Following publication of an online accepted manuscript, several readers pointed out aspects of the research design and limitations in the statistical analyses that undermined the authors’ statements about the intervention studied. The editors upheld these concerns and subjected the online manuscript to statistical review, which confirmed the need for significant revisions. The authors agreed to make substantial changes to the manuscript before the publication of a final published version.

These included changing the title, addressing faults in the research design and aims, results, and conclusions, adding further information about the ethical approval of the study and the addition of a link to supplementary material. There is now the addition of discussion of the significance of femoral neck versus total hip bone mineral density measurements; removal of data about biomarkers; replacement of the prior table 2 with a table that includes non-parametric tests with Bonferroni correction; the addition of a table that provides coefficients and p-values of the regression model applied for each response separately; the addition of a table that describes statistics at baseline and 12 months later; the addition of the new references 4, 5, and 6.

